# Faculty Development for Academic Emergency Physicians: A Focus Group Analysis

**DOI:** 10.7759/cureus.27596

**Published:** 2022-08-02

**Authors:** Kiran Pandit, Wendy C Coates, Deborah Diercks, Sanjey Gupta, Jeffrey Siegelman

**Affiliations:** 1 Emergency Medicine, Columbia University, New York City, USA; 2 Emergency Medicine, University of California, Los Angeles David Geffen School of Medicine, Los Angeles, USA; 3 Emergency Medicine, University of Texas Southwestern Medical Center, Dallas, USA; 4 Emergency Medicine, Zucker School of Medicine, Hempstead, USA; 5 Emergency Medicine, Emory University, Atlanta, USA

**Keywords:** academic society, qualitative research, emergency medicine, professional organization, faculty development

## Abstract

Objectives: The objective is to explore academic emergency medicine physicians’ exposure to and needs regarding faculty development.

Methods: We conducted a prospective qualitative study of Society for Academic Emergency Medicine members in 2018 using focus groups selected by convenience and snowball sampling. One facilitator ensured representative engagement and responses were transcribed in real-time by an assistant after obtaining verbal consent. Results were analyzed using a grounded theory approach with a constructivist perspective. Thematic analysis was refined using the constant comparative method.

Results: Sixteen physicians participated in the focus groups, representing a diverse group of perspectives. Six themes emerged about unmet needs in faculty development: knowledge and skills, relationships, specific programs or resources, and professional benefits.

Conclusions: Members of a national academic society identified three areas of focus important to developing academicians in emergency medicine: content for faculty developers, relationship-building among members, and support from the organization as a “professional home.” Academic societies can use this to guide future programming.

## Introduction

Faculty development in academic medicine refers to all activities health professionals pursue to improve their knowledge, skills, and behaviors [1]. Professionals desire such avenues to be more effective in their work, and to be more productive academically. This is essential to having a satisfying, successful career in academic medicine [2,3].

The field of faculty development has rapidly expanded over the last two decades [1], growing beyond the traditional foci of academic effectiveness [4] and promotion [5]. Faculty development now more broadly refers to an academician’s overall opportunities for personal and professional growth [1]. As a result of many needs assessments over the years [3,6,7], faculty development activities now encompass a varied array of topics such as continual professional development (CPD), including clinical skill maintenance [8], scientific writing [9], scholarship [10], research [11], leadership and management [12], physician wellness [13], mentorship [2], development of a professional identity [14], and more.

This trend of expanding the breadth of faculty development is also seen within the field of Emergency Medicine (EM). Emergency physicians have expressed a strong desire for diverse skills, such as research, teaching, communication [3], leadership [15], physician wellness, and mentorship. In addition, there is a need for awareness of existing faculty development resources [13]. With the expansion in topics has come a growth in the target audience of faculty development activities, from its initial emphasis on new faculty to an increasing understanding of the needs of mid-career faculty, senior faculty, and department chairs [16-18]. There is also an emphasis on expansion beyond physician-educators with major teaching roles, to medical education researchers and faculty developers [19], and those who focus on scientific research, administration and leadership, and advocacy. Expansion of faculty development opportunities has led to a new emphasis on ensuring the effectiveness of faculty development programs via measurement of specific outcomes and impact [4,20-22].

The literature reflects a nascent interest in the subject of faculty developers as a group of educators and learners themselves. There is sparse research on the identity formation and motivations of faculty developers [23,24], but many questions remain about their roles and responsibilities and how they can best conduct faculty development activities. In addition, there is a growing expectation that faculty developers undergo formal training in how to conduct faculty development in a scholarly manner [18]. Although there are many faculty development initiatives that target specific groups, such as junior faculty at an individual university, there is no information to direct leaders in EM who wish to create faculty development initiatives for the EM academic community at large.

Previously, the organizational structure for faculty development activities was largely based in educational institutions such as universities and medical schools [25], but is now moving to academic departments and associations of higher education [7,18]. Aware of this trend, the Society for Academic Emergency Medicine (SAEM), as an association focused on the development of academic emergency physicians, aims to provide faculty development programming for its members. An increasing number of SAEM members are not affiliated with a university or medical school [26]. The question remains, “What is the best way for a national organization to provide faculty development programs for its members?” To answer this question, SAEM was selected as an example of a professional organization, but the lessons learned from studying this question are applicable to many academic organizations.

In order to effectively drive future organizational programming, the SAEM Faculty Development Committee sought to understand academic emergency physicians’ exposure to and needs regarding faculty development initiatives, within the context of broad trends in the field of faculty development.

## Materials and methods

Setting and selection of participants

Participants were selected by convenience sampling and snowball sampling, beginning with the identification of potential subjects by members of the SAEM Faculty Development Committee and the Board of Directors. These members were encouraged to recruit other suitable participants. To expand diversity, a recruitment email was sent to all SAEM members. Each subject was enrolled by a personal email with standard text describing the project’s objectives, focus group process, and how responses would be handled. 

Study design

This was a prospective qualitative study that was conducted in a hotel conference room in San Diego, CA, concurrent with the ACEP Scientific Assembly in October 2018. Three discrete one-hour focus groups were facilitated by a mid-career academic emergency physician (JS) who has training in the use of focus groups for qualitative research. The focus groups were audio recorded and transcribed by a neutral administrative assistant provided by SAEM. Using a predetermined template, the facilitator guided the discussion, ensuring widespread participation by all members, and performed member checking to ensure understanding of the intended meaning in real-time. We determined that each focus group would consist of a varied sampling of participants. The Institutional Review Board at the Emory University School of Medicine deemed the study non-human subjects research, and thus exempt from requiring full IRB review. All participants consented to an audio recording of the sessions and dissemination of findings with anonymity.

Development of instrument

All authors participated in the development of questions to be posed during the focus group, including introductory discrete questions to discover SAEM members’ exposure to and needs regarding faculty development initiatives. Item clarity was optimized by reading the script aloud and modifying the questions on the agenda. We constructed open-ended questions to allow respondents to reflect on their individual experiences and welcomed natural discussion responses from other members of the focus groups. Questions are listed in Table 1.

**Table 1:** Questions posed to the focus group participants

Questions posed to the focus group participants
Where do you see yourself in five years? How will you get there? What will you need to make this happen? For senior faculty: Are you now where you thought you wanted to be five years ago? How did you accomplish your progress? What would have made it easier?
How would you define faculty development, as it relates to a national society?
What faculty development opportunities are lacking at your institution that SAEM can provide?
What changes to SAEM’s online content would be helpful for your own faculty development?
Would a faculty development textbook/manual be useful to you? If so, what should be in it?

Data analysis

We used grounded theory with a constructivist perspective to analyze the focus group discussions. Using the constant comparative method, we refined our thematic analysis using an inductive process to identify broad themes and sub-themes. The transcripts for all three focus groups were distributed to all authors, giving each author the opportunity to review the transcripts. Three investigators, who have experience in qualitative coding methods (DD, SG, and KP), independently coded each transcript. All authors discussed the preliminary coding scheme and one author (KP) then generated a streamlined coding template which was then applied by the original coders. All authors met to discuss and resolve disputes. One author (SG) who participated as a focus group member was excluded from coding the resulting transcript, which was then coded by a substitute (WC).

## Results

In addition to the focus group moderator and SAEM staff, 16 SAEM members participated in the focus groups. Table 2 shows the demographics of focus group participants.

**Table 2:** Focus group participant demographics

Characteristic	Number (%)
Gender	
- Male	12 (71)
- Female	5 (29)
Rank	
- Assistant Professor	8 (47)
- Associate Professor	7 (41)
- Professor	2 (12)
United States Geographic Region	
- Northeast	5 (29)
- Southeast	3 (18)
- Midwest	2 (12)
- West	7 (41)

We identified three major themes. The first was the need for specific knowledge and skills, the consumers of which can be divided into two target groups: academic EM physicians (for growing their own careers) and faculty developers (for growing the careers of others). Academic EM physicians were interested in knowledge and skills regarding clinical care, research, education, finance, leadership, and management. Faculty developers expressed a need for knowledge and skills in how to conduct faculty development activities effectively. Both groups expressed concern about a lack of awareness of existing resources related to faculty development, as members found resources in various locations by talking to other members, rather than through an organized repository of relevant resources.

The second major theme dealt with relationships; many focus group participants discussed how their career development was impacted by their relationships with peers and more senior mentors. These relationships were important for networking to exchange ideas and make professional connections, and also for accessing experienced mentors and coaches. In general, participants felt they would benefit from better mechanisms for connecting with other academic emergency physicians and for mentorships, such as virtual portals or online meeting sites.

The third major theme was a range of professional benefits that impact faculty development work. One sub-theme was the value of faculty development offerings in terms of financial cost, time spent, and investment of effort and attention. Another sub-theme was support from professional institutions, including academic or hospital departments, and national organizations, to pursue career growth, as well as a sense that this support created a sense of a “professional home.” Table 3 shows representative quotes within each theme.

**Table 3:** Representative quotes by theme

Theme	Sub-Theme	Representative Quotes	
Knowledge & Skills	Knowledge and Skills for academic EM physicians: clinical skills, finance skills, leadership skills	“Some skill training is needed for older faculty that don’t know newer processes.” “I feel like my weaknesses are budgets, finances, and larger healthcare movement issues.” “SAEM doesn’t have a structured way to provide an individual with faculty development consultation.” “I did the Chair Development Program at SAEM. I felt it was incredible (especially in training how to do faculty development). I also did the Harvard 2-week Chair Development Program.”	
	Knowledge and Skills for faculty developers: best practices and guidelines for conducting faculty development	“There is no step by step guide for an ideal faculty development program. Maybe SAEM could publish one?” “The Faculty Development program was handed to me as an Associate Professor and Vice Chair without any training on what to do.”	
	Lack of awareness of information/opportunities	“Many members don’t know what information is on the website and so can’t take advantage of what’s there.” “A common problem is that the general population doesn’t know how to access resources. The general membership doesn’t see all the work that SAEM does.”	
Relationships	Making connections and networking Community for idea exchange	“Perhaps a network to go to depending on the career path or skill set you want to develop would be helpful.” “You really form relationships when you can collaborate on a project together.”	
	Access to leaders, mentorship, coaching	“A lot of places don’t have great mentoring mid-career.” “Taking advantage of interest groups and academies is the best way to get mentorship from senior faculty from other institutions.” “It would be interesting to have a Match.com type database for mentorship to see where each person’s area of expertise is.”	
Professional benefits	Value for time/money/attention	“One of my faculty members asked me if it was worth it to join SAEM and I didn’t know what to tell them.” “Making interest groups and academies free of cost will help encourage people to choose more than one group and allow for cross-pollination.” “Don’t drive up the cost of general membership by adding things not everyone may use. There should be a tiered cost depending on what types of things you would like to add to your membership. Otherwise, people may be disincentivized from joining SAEM. The cost of time should be considered as well.”	
			Institutional support Professional home	“I’m looking to find a ‘home’ in interest groups but haven’t found it yet.” “I feel lucky that my institution is very supportive…. I have been in several programs through my institution (teaching/mentoring academy, clinical Teaching Scholars Program).” “I feel like SAEM is my professional home.”	

## Discussion

We found that faculty members expressed a need for cost-effective faculty development initiatives in a variety of areas of knowledge and skills. They also desired to strengthen and deepen professional relationships, bring long-term benefits to their careers, and to help meet professional goals. By working to meet these needs, an academic society can serve as a professional home for more faculty. These findings support and build upon prior literature and suggest several recommendations for SAEM and other similar academic societies.

Focus group participants expressed a need for traditional faculty development initiatives to grow their own careers. In some cases, this need arises when members’ needs are not being adequately addressed by local institutions. This may be due to the number of members who are not located at major university settings [26], because members are unaware of or unable to access existing resources [13], or because institutional faculty development programs are often not specific to learning and teaching in the emergency department workplace [1,19]. As a result, the locus of faculty development activity is shifting from medical schools to local academic departments and professional associations [7,18]. Those responsible for delivering faculty development content locally spoke to a need to also have content specifically for them.

The focus groups also highlighted the importance of personal relationships to faculty development. Academic societies may be most effective focusing on collaborative relationships or communities of practice as mechanisms for members’ professional growth [19,27]. Supportive professional relationships and intentional community building are critical to effective faculty development [1,21]. High-quality mentorship is critical for the professional success of academic EM physicians [28], though this need remains unmet for many [13,29].

At the same time, there are practical considerations for the success of faculty development initiatives. Junior faculty have named the availability of time and time management as a top barrier to achieving their professional goals [30]. These barriers and others (financial, clinical obligations, etc.) provide both topics for content and important areas of focus in designing faculty development opportunities. Recommendations for addressing these issues are presented in Table 4.

**Table 4:** Recommendations for Academic Societies’ Faculty Development Initiatives

Theme	Specific recommendations
Important content	Develop curricula targeted to each career stage. Focus on groups with unmet needs. Provide content specific to faculty developers, including curriculum design and program assessment.
Relationships	Leverage technology to increase opportunities for members to connect, collaborate, and support each other. Encourage connections between groups. Develop a matching process for connecting mentees with mentors with relevant interest and expertise. Foster relationships through interest group and committees.
Logistics	Consider cost, location, and time in planning faculty development activities. Leverage video conferencing when appropriate. Align learning objectives of the activity with format and logistical needs.
Professional home	Identify members’ needs which are unmet at their local department or institution. Develop products and opportunities unique to the society.

Limitations

Our findings are limited by several factors. The number of focus groups conducted was small, with uneven numbers of participants. Focus group participant demographics skewed male and with few senior faculty. As such, the views expressed may not be completely representative of the needs and experiences of the entire society’s membership. In addition, the views of the society’s members may not reflect those of all academic EM, especially those from other nations. A more robust sample may have led to different findings. A future study might include a focus on subgroups such as mid-career faculty, and those under-represented in EM.

The focus group sessions were transcribed by an independent transcriptionist using summative transcription, rather than court-style transcription with exact quotes, which could potentially have introduced the transcriptionist’s interpretation or summarization of comments. Inherent in this type of qualitative research is the possibility of personal bias and misinterpretation of participants’ words. 

## Conclusions

Our study highlights the core faculty development needs of members of one academic society, namely: content for both academic physicians and faculty developers, relationship-building among members, and support from the organization as a “professional home.” These findings can inform the development of organizational programming to address these needs. Other professional organizations may find that our recommendations for faculty development initiatives may apply to the needs of their members, as well.
